# An automatic tooth preparation technique: A preliminary study

**DOI:** 10.1038/srep25281

**Published:** 2016-04-29

**Authors:** Fusong Yuan, Yong Wang, Yaopeng Zhang, Yuchun Sun, Dangxiao Wang, Peijun Lyu

**Affiliations:** 1 Centre of Digital Dentistry, Peking University School and Hospital of Stomatology & National Engineering Laboratory for Digital and Material Technology of Stomatology & Research Centre of Engineering and Technology for Digital Dentistry of Ministry of Health, 22 Zhongguancun Nandajie, Haidian District, Beijing, 100081, China; 2 State Key Lab of Virtual Reality Technology and Systems, Beihang University, 37 Xueyuan Road, Haidian District, Beijing, 100191, China

## Abstract

The aim of this study is to validate the feasibility and accuracy of a new automatic tooth preparation technique in dental healthcare. An automatic tooth preparation robotic device with three-dimensional motion planning software was developed, which controlled an ultra-short pulse laser (USPL) beam (wavelength 1,064 nm, pulse width 15 ps, output power 30 W, and repeat frequency rate 100 kHz) to complete the tooth preparation process. A total of 15 freshly extracted human intact first molars were collected and fixed into a phantom head, and the target preparation shapes of these molars were designed using customised computer-aided design (CAD) software. The accuracy of tooth preparation was evaluated using the Geomagic Studio and Imageware software, and the preparing time of each tooth was recorded. Compared with the target preparation shape, the average shape error of the 15 prepared molars was 0.05–0.17 mm, the preparation depth error of the occlusal surface was approximately 0.097 mm, and the error of the convergence angle was approximately 1.0°. The average preparation time was 17 minutes. These results validated the accuracy and feasibility of the automatic tooth preparation technique.

Tooth preparation, which is a basic aspect of the treatment of hard-tissue dental diseases, is the process used for the quantitative preparation and formation of hard tissues on a patient’s diseased teeth. At present, tooth preparation is generally performed with a high-speed dental handpiece. However, this method has a number of disadvantages. First, these procedures depend on the accuracy of human vision and movements in the narrow space of the oral cavity (generally 2.5–5.0 cm length of mouth opening). Dentists need to avoid injuring the muscles and soft tissues of the tongue, lips and cheeks because random jaw motions can be encountered during the tooth preparation process. Achieving high tooth preparation standards, such as an accurate convergence angle and small occlusal and axial reduction amounts, is a challenging task for manual operations. As a result, teeth are commonly over- or under-prepared, which influence restoration quality, even cause iatrogenic injury. Excessive removal of tooth structure can make a tooth overtapered or shortened too much, and thus cause an unnecessary sacrifice of retention and resistance. Thermal hypersensitivity, pulpal inflammation, and necrosis can result from approaching the pulp too closely. Under preparation can lead to insufficient restoration space, which results in over contour of the gingival third of the restoration and that may lead to soft tissue damage. Second, the required dental handpiece generates high-pitched noises and vibrations that make both the patient and dentist uncomfortable^1^,^2^,^3^.

Laser preparation of dental hard tissue is considered to be safer and more comfortable compared to the use of a traditional handpiece because it produces less pain and reduces noise and vibrations^4^. In recent years, ultra-short pulsed lasers have been introduced in restorative dentistry to overcome the drawbacks of traditional treatment methods. The so-called ultra-short pulse laser (USPL) has picosecond (1 picosecond = 10^−13^ seconds) and femtosecond (1 femtosecond = 10^−15^ seconds) pulse widths^5^,^6^. These laser pulses, which are amplified with energies of up to millijoules^7^ and are focused on the material’s surface, allow thin layers to be ablated with a high accuracy and reproducibility, which can result in much less collateral damage to the adjacent tissues compared to using other thermal, chemical, or mechanical processes^8^,^9^. Krüger *et al.* pioneered the study of using femtosecond lasers in restorative dentistry^10^. Daskalova *et al.* demonstrated that by selecting suitable parameters, one could obtain efficient dentin surface preparation without evidence of thermal damage, i.e., with minimised heat-affected zones and reduced collateral damage^11^. Such merits could make USPL technology a good candidate for treating dental hard tissue with accurate preparation outlines and a lack of thermal damage around the prepared cavities. However, if the USPL is controlled by hand to complete the tooth preparation process, the accuracy requirements are still difficult to achieve.

In the last 20 years, medical robotic technology has been widely studied with the aim of overcoming the limitations of manual operation and improving the accuracy and stability of surgical operations^12^,^13^,^14^,^15^,^16^,^17^. Compared to manual operation, robotic operation can provide potential benefits, including motion planning based on three-dimensional (3D) digital models to achieve a higher accuracy, stability to avoid trembling of the human hand and efficiency to reduce operation time. Furthermore, robotic manipulation may relieve the fear and discomfort associated with conservative dental treatment^18^ and can provide high-accuracy performance through carefully designed sensors and actuation systems in various surgical operations^19^. However, according to reports in literature, there has been no reported study that combines laser technology and medical robotics for clinical 3D tooth preparation operations.

To overcome the drawbacks of current manual tooth preparation techniques, in this study, an automatic tooth preparation system was developed. This system aims to precisely control a USPL beam for preparing tooth hard tissues and thus to replace conventional mechanical grinding equipment. Preliminary experimental results on preparing 15 first molars in a phantom head validated the accuracy, efficiency and safety of the developed system.

## Materials and Methods

### Components of the robotic system

**Figure 1:**
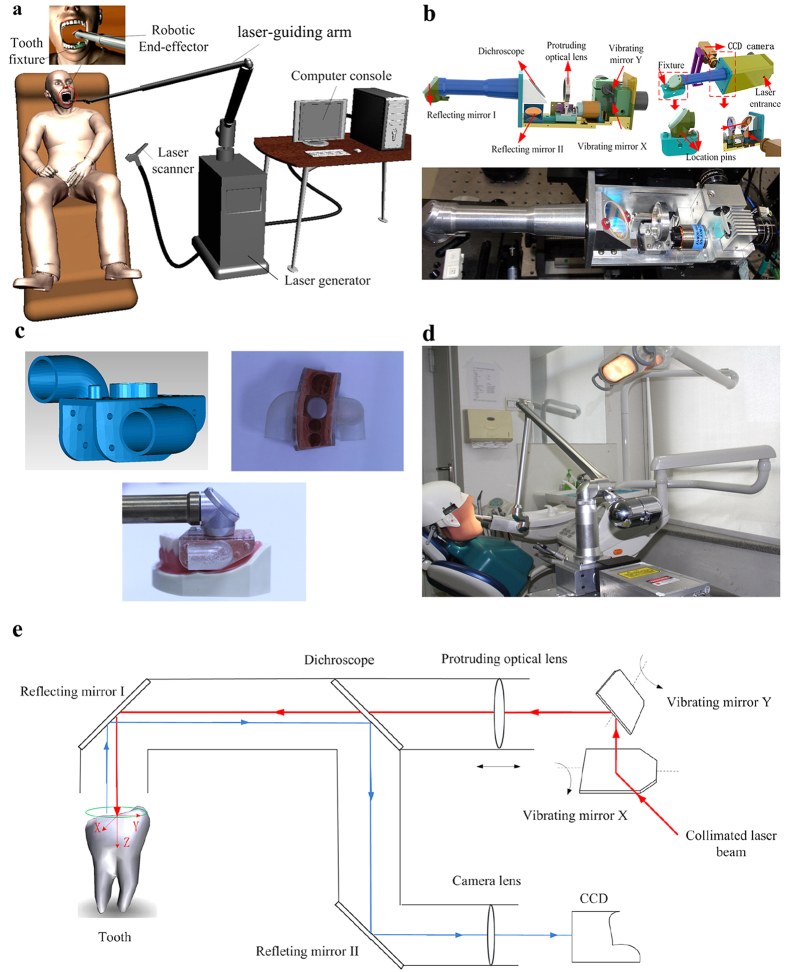
Components of the automatic full crown tooth preparation system. (**a**) Conceptual schematic diagram of the automatic full crown tooth preparation system. (**b**) Physical prototype of the robotic device that was used to perform tooth preparation. (**c**) Physical prototype of the tooth fixture and the connections among the device, tooth fixture, and the target tooth. (**d**) Design of the intraoral automatic tooth preparation system. (**e**) Diagram of the system’s working principle.

**Figure 2:**
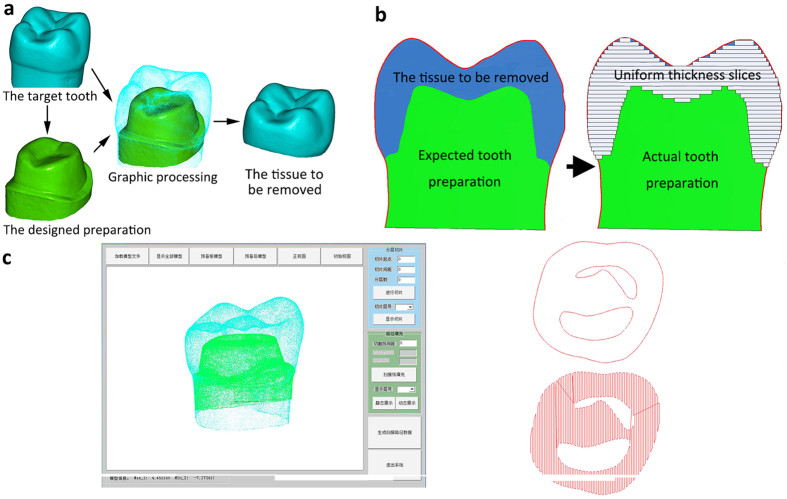
Preparation of the tooth for the automatic technique. (**a**) Computer-aided design (CAD) of the tooth to be prepared for a full crown and the three-dimensional (3D) data for the dental tissue requiring removal. (**b**) Schematic diagram of the preparation of the tooth for the layered preparation. (**c**) The numerical control software and the design of the path for laser scanning for the single-layered preparation.

**Figure 3:**
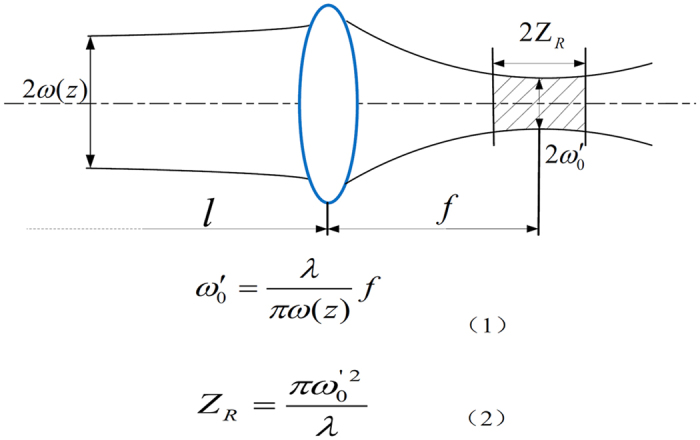
Mathematic model of the Gaussian laser-beam distribution used in the development of the automatic laser-control micropreparation unit in which *ω(z)* is the section radius of the incident Gaussian beam, which is related to the propagation distance of the light beam; *λ* is the wavelength of the light beam; *f* is the focal length of the lens; *ω′*_*0*_ is the waist radius of the emergent light beam; and *Z*_*R*_ is half of the emergent light beam’s waist length, which is the effective working length for laser preparation.

According to the research ideas and design principles (Fig. 1a), the system was developed with the following hardware components: 1) an intraoral 3D scanner (TRIOS, 3Shape A/S, Copenhagen, Denmark) to obtain the 3D data of the patient’s target tooth, adjacent teeth, opposing teeth and the teeth fixture; 2) a computer-aided design (CAD) (Fig. 2a)/computer-aided manufacturing (CAM) (Fig. 2b,c) software for designing the target preparation shape and generating a 3D motion path of the laser; 3) an effective low-heat USPL generator to provide USPL beams that are suitable for hard tissue preparation; 4) a six-degrees-of-freedom light guiding arm that can easily guide the laser beam toward the robotic device, resulting in improved collimation and flexibility of the laser conduction (if the patient moves slightly, the device can synchronously move with the system, which ensures the flexibility of the system); 5) a robotic system (i.e., an intraoral automatic laser-controlled micro-preparation unit) (Fig. 1b) that comprises two high-speed galvanometers and one focusing lens, designed according to dental ergonomics and the Gaussian laser-beam distribution model (Fig. 3) (the system produces a high speed (1,900 mm/s), high accuracy (step precision, 1 μm), and lens focal length (175 mm); the laser beam transmission path is shown in Fig. 1e); and 6) a tooth fixture that not only connects the robotic device with the target tooth but also protects the adjacent teeth from laser cutting (the tooth fixture was designed using Solidworks software (Dassault Systèmes SOLIDWORKS Corporation, Waltham, MA, USA) and was manufactured with the 3D printing method with an accuracy of 20 μm (Fig. 1c)). By integrating the six components described above, the entire system could meet the requirements of intraoral automatic tooth preparation (Fig. 1d).

### Additional experimental equipments

Using the developed tooth preparation system, automatic full crown tooth preparation experiments were performed within a phantom head.The following additional equipments were used for the tooth preparation experiment: a Q-switch mode-locked all-solid-state picosecond laser generator (1,064 nm wavelength, 20 ps pulse width, 100 KHz repetition rate, 30 W power, and 38 μm spot diameter), a phantom head (Nissin, Tokyo, Japan), an intraoral scanner (TRIOS, 3Shape A/S, Copenhagen, Denmark), CAD software for tooth preparation for a full metal crown, Geomagic software (Studio and Qualify 2012, 3D Systems, Inc., Rock Hill, SC, USA), and Imageware (13.1, Siemens PLM Software, Berlin, Germany).

### Collection and pre-processing of the tooth samples

A total of 15 maxillary and mandibular intact first molars that were freshly extracted at the Hospital of Stomatology in Peking University were collected. The study was approved by the Bioethics Committee of the Stomatological Hospital of Peking University (Beijing, China; No. PKUSSIRB-201405008a; Date: 08/05/2014). All of the experimental protocols and procedures were approved by the licensing committee and performed in accordance with the approved guidelines and regulations. The patients were informed that the extracted teeth would be used in the *in vitro* study, and informed consent was obtained from all of the subjects. An ultrasonic scalar was used to remove dental calculi and soft tissues from the surface of the removed teeth, which were then rinsed with physiological saline.

### Designing the 3D target shape of the prepared teeth

The phantom head was fastened on the dentistry complex therapy chair and maintained in the clinical tooth preparation position by adjusting the dentistry chair. First molars were removed from the standard artificial teeth model. Then, one of the fifteen study teeth was fixed onto the corresponding tooth bit of the standard artificial teeth model, and an intraoral scanner was used to obtain 3D data (data I) of the prepared teeth *in vitro* and the standard artificial teeth model. The tooth fixture was fastened on the prepared tooth and its adjacent teeth with an amorphous silicone rubber seal that helps to retain and protect the adjacent teeth and gingival soft tissue during treatment. Then, the intraoral scanner was used again to obtain 3D data (data II) for the prepared teeth *in vitro*, the standard artificial teeth model and the tooth fixture. The data I and data II were registered into one coordinate by a common area registration method using the Geomagic Studio software. The target preparation shapes of these molars were designed using customised CAD software.

### Automatic tooth preparation process

The rear part of the tooth preparation robot was connected with the six-degree-of-freedom light guiding arm, and the head part of the robot was rigidly docked with the tooth fixture by means of a standard mechanical interface. Through the registration mast of the tooth fixture, the robot and prepared tooth were aligned precisely in a coordinate system. With this component, the robotic device was fixed in the mouth. The 3D data of the tooth before preparation and the CAD data of the prepared tooth were inputted into the numerical control software to obtain 3D data of the tissues requiring removal. A 46-μm single-layer preparation depth and 500 total preparation layers were adopted to complete discrete layered sections of the 3D CAD model of the prepared teeth. Scanning line filling and scanning path planning were performed according to the section’s outline information, and selective preparation of the tooth surface was performed by the robot controlling the picosecond laser to obtain a 2D-plane image of a specific layer. This process was completed by changing the direction of the high-speed 2D galvanometers. Five layers of pulses and a 46-μm single-layer preparation depth were the settings for the convex stepping, such that the convex moved forward by 46 μm as the laser scanned five layers of pulses on the tooth surface and completed the preparation of five layers of the section outline. This step was followed by another round of sectioning and scanning preparation. This process continued until a 3D morphology of the tooth preparation was generated. During the whole process, a timer was used to record the preparation time of each tooth.

### Evaluation of tooth preparation accuracy

#### The overall shape error analysis

**Figure 4:**
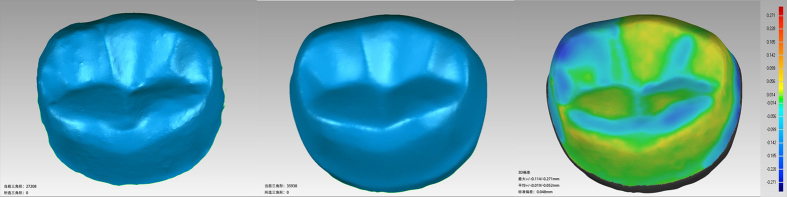
Software analyses of the 3D data for the preparation results and the CAD data for the prepared teeth.

The 3D data of each tooth after preparation were obtained with the intraoral 3D scanner, and the data were compared to the original CAD data of the target shape to obtain the overall shape error using the Geomagic Qualify software. The mean error was calculated (Fig. 4).

#### The occlusal reduction depth and convergence angle error

**Figure 5:**
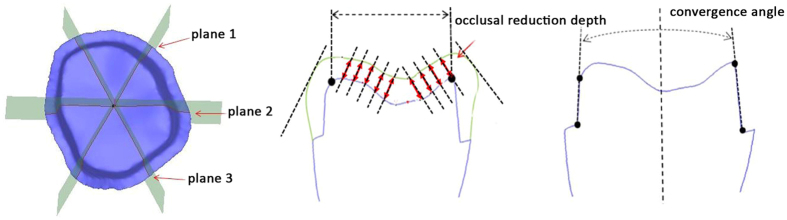
Schematic diagram of the measurement methods to determine the occlusal reduction depth and the convergence angle of the preparation results.

The occlusal reduction depth and convergence angle error are the main indexes for clinically evaluating the tooth preparation quality. The 3D data of the prepared teeth were obtained with an intraoral 3D scanner and imported into the Imageware software along with the 3D data of the intact first molar. The common area registration method was then used to register the two sets of data into the same coordinate system. Then, three virtually vertical planes in the buccolingual (b-l) (plane 2), mesiobuccal–distolingual (mb–dl) (plane 1), and distobuccal–mesiolingual (db–ml) (plane 3) directions crossing one central point and forming horizontal tapers of 60° under each other were manually constructed. Three 2D cross sections were generated. In the b-l cross section, the occlusal reduction depth was assessed by measuring 10 points on the occlusal surface. Consequently, 150 values (10 points × 15 teeth) were measured. The mean values for each prepared molar were calculated. The convergence angle was determined by analysing the taper between two opposing surfaces of all three planes. Consequently, 45 values (3 × 15) were measured. The mean values for each group were calculated (Fig. 5). The results were analysed with SPSS 20.0 (IBM Corporation, Armonk, NY, USA). One-way analysis of variance (ANOVA) was used to evaluate the variance of the occlusal reduction depth and convergence angle among different samples.

## Results

### Accuracy of the automatic tooth preparation technique

**Figure 6:**
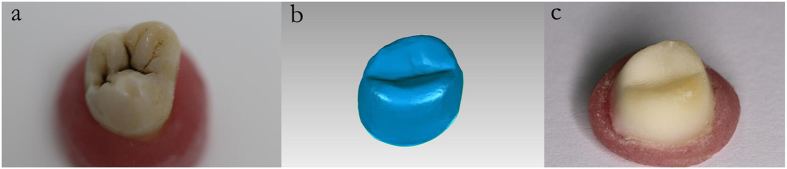
Preparation results. (**a**) The first molar; (**b**) 3D model of tooth preparation by CAD; (**c**) The result of automatic tooth preparation.

**Figure 7:**
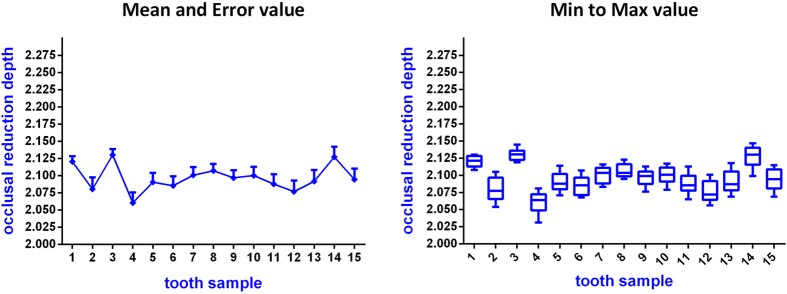
Statistical diagram of the occlusal reduction depth measurement of 15 tooth samples.

**Figure 8:**
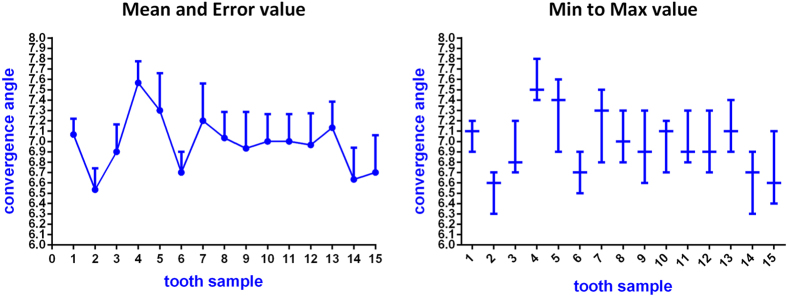
Statistical diagram of the convergence angle measurement of 15 tooth samples.

Fifteen full crown tooth preparations with complete and smooth occlusal and axial surfaces were obtained during the experiment (Fig. 6). The average time required for each preparation process was 17 min. Compared with the CAD data of standard tooth preparation, the overall average errors assessed with the Geomagic studio for the 15 prepared teeth showed a range of 0.05–0.17 mm. As shown in Table 1, the occlusal reduction depths of the 15 preparations were greater than 2 mm; the maximum was 2.147 mm, the minimum was 2.031 mm, the average value was approximately 2.097 mm, and the mean error measured with the software was approximately 0.097 mm for the occlusal reduction depth (Fig. 7). Using one-way ANOVA analysis, the occlusal reductions differed significantly among the different samples (P < 0.05) (Table 2). In addition, the 45 measured convergence angles showed a maximum of 7.8°, a minimum of 6.3°, a mean value of approximately 7.0°, and a mean error of approximately 1.0° (Table 3) (Fig. 8). In addition, the convergence angle differed significantly (one-way ANOVA, P < 0.05) among the samples (Table 4).

## Discussion

### The role of the phantom head

In this study, automatic robotic control technology was used to develop an automatic full crown tooth preparation system. The entire tooth preparation process was performed within the phantom head with a numerically controlled laser beam. The phantom head played an important role in the study, which made the whole experiment more similar to the clinical setting. First, the phantom head with a maxillary and mandibular standard dental model simulates the narrow oral space, the small distance between the up and down occlusal plane, and the relative position among the dentition, tongue palate, and lip cheek. These similarities helped to verify the suitability of the tooth preparation robotic device used in the oral cavity, e.g., whether the device could be efficiently mounted and dismounted. Second, the phantom head fixed on the dentistry complex therapy chair can simulate the patient’s body position during tooth preparation. Third, the phantom head can imitate a variety of complex movements of the patient’s head. The feasibility for automatic tooth preparation with an integrated laser manipulation and electrical motor devices was verified, and preliminary experimental results confirmed that the proposed system has the potential to meet the clinical requirements of tooth preparation.

Based on the experimental results, several limitations of the current system and future research topics were identified.

### Preparation accuracy

Although the automatic tooth preparations met the clinical requirements in the visual inspections and software analyses in the study, only the preparation of non-shouldered full crowns was achieved thus far. Other types of restorations, such as prepared whole shapes, inlays, veneers, partial crowns, and implant osteotomy of decayed teeth, require different preparation quantity and preparation methods. Variations in the structure and composition of teeth that are found among different age groups, individuals, teeth positions, and even parts of the same tooth, as well as in the resin and metal fillings frequently found in the teeth to be prepared, all affect the accuracy of automatic tooth preparations. In future studies, the laser parameters will be optimised, the laser preparation depth and convergence errors on hard dental tissues will be studied and analysed, and an accurate mathematical model will be established. At the same time, the feasibility of the use of different tissues and materials will be studied.

### Preparation efficiency

To achieve automatic control of tooth preparation, an algorithm was developed to define the 3D moving path of the laser focal point on the surface of the target tooth^20^. Obviously, the selection of a different moving path could lead to a different preparation efficiency. In current system, it was observed that a layered preparation method was selected to perform the entire automatic tooth preparation process. This method can obtain a high accuracy; however, the time cost for preparing a tooth crown was approximately 17 minutes. In future work, we plan to observe the effect of a volume preparation method. Compared with the layered preparation method, the volume preparation method aims to ablate a 3D volume from the tooth instead of ablating layer by layer. This new method might decrease the amount of dental tissue removed during tooth preparation, thus increasing the efficiency of automatic tooth preparation. However, compared with layered tooth preparation, there is light blocking during preparation with the volume cutting method; in other words, the laser beam cannot access the bottom of the target volume. Therefore, it is an open challenge to propose an efficient preparation method while avoiding the light blocking phenomenon.

Different laser control parameters, such as the surface scanning rate and energy density, also affect the preparation efficiency^21^. In this study, USPLs with different energy densities were used for hard-tissue preparation at different scanning rates, and these lasers were controlled by a robot. The results for the preparation efficiency showed that the maximum preparation efficiency and best preparation quality were achieved at an energy density of 4.42 J/m^2^ and scanning rate of 1,900 mm/s. In the study, the ranges of scanning rate and energy density observed during the experiment were limited. Better parameter combinations beyond the observed range that can further increase the preparation efficiency are possible. In addition, the effects of other related parameters, such as wavelength, pulse width, frequency, pulse shape, and focal position, on the preparation efficiency of the USPL also require further investigation.

### Preparation safety

Safety is a primary concern in the clinical application of robotic automatic control. In this study, the robotic device was connected in a stable manner to the target tooth crown with a tooth fixture, and this ensured that the robotic device was always in the same coordinate system as the target tooth and protected the surrounding soft and hard tissues of the target tooth crown from injury. To enhance the safety performance of the entire system, the effect of real-time monitoring of the charge-coupled device in three dimensions and a rapid connect/disconnect device will be studied in future research so that the planned effects of the 2D plane as well as the planned conditions of the axial plane and vertical direction can be observed on the external display. As a result, the system can be quickly stopped under any emergency situation, thus ensuring the safety and controllability of the entire system.

Further improvements in this technique are expected to transform and innovate oral clinical operation techniques from reliance on visual inspections and manual operations to artificial intelligence and automation.

## Conclusions

In this study, a novel concept of automatic dental preparation solution using multi-disciplinary knowledge consisting of robotics, ultra-short wavelength laser, 3D motion control, and oral medical technology were proposed for the first time. The current experimental results in the phantom head preliminarily illustrated the potential of the automatic tooth preparation technique for use in dental clinics, and the accuracy of automatic tooth preparation met the accuracy requirements of tooth crown preparation defined in dental textbooks.

## Additional Information

**How to cite this article**: Yuan, F. *et al.* An automatic tooth preparation technique: A preliminary study. *Sci. Rep.* **6**, 25281; doi: 10.1038/srep25281 (2016).

## Figures and Tables

**Table 1:** The statistical description of the occlusal reduction depth measurements.

	N	Mean	Std. Deviation	Std. Error	Minimum	Maximum
1	10	2.12030	0.007959	0.002517	2.108	2.130
2	10	2.08030	0.017121	0.005414	2.054	2.105
3	10	2.13010	0.008425	0.002664	2.119	2.145
4	10	2.06040	0.014998	0.004743	2.031	2.081
5	10	2.09040	0.013729	0.004342	2.071	2.114
6	10	2.08520	0.013839	0.004376	2.068	2.107
7	10	2.10040	0.012112	0.003830	2.083	2.116
8	10	2.10710	0.010038	0.003174	2.095	2.123
9	10	2.09670	0.011076	0.003503	2.076	2.113
10	10	2.10010	0.012940	0.004092	2.079	2.117
11	10	2.08760	0.014315	0.004527	2.065	2.113
12	10	2.07670	0.016042	0.005073	2.056	2.101
13	10	2.09160	0.016453	0.005203	2.069	2.118
14	10	2.12710	0.015096	0.004774	2.099	2.147
15	10	2.09430	0.015798	0.004996	2.069	2.115
Total	150	2.09655	0.022444	0.001833	2.031	2.147

Note: The standard prepared occlusal reduction depth was 2 mm.^22^

**Table 2:** One-way analysis of variance of the occlusal reduction depth measurements.

	Sum of Squares	dof	Mean Square	F	P value
Between Groups	0.050	14	0.004		
Within Groups	0.025	135	0.000	19.279	0.000
Total	0.075	149			

dof, degrees of freedom.

**Table 3:** The statistical description of the convergence angle measurements.

	N	Mean	Std. Deviation	Std. Error	Minimum	Maximum
1	3	7.067	0.1528	0.0882	6.9	7.2
2	3	6.533	0.2082	0.1202	6.3	6.7
3	3	6.900	0.2646	0.1528	6.7	7.2
4	3	7.567	0.2082	0.1202	7.4	7.8
5	3	7.300	0.3606	0.2082	6.9	7.6
6	3	6.700	0.2000	0.1155	6.5	6.9
7	3	7.200	0.3606	0.2082	6.8	7.5
8	3	7.033	0.2517	0.1453	6.8	7.3
9	3	6.933	0.3512	0.2028	6.6	7.3
10	3	7.000	0.2646	0.1528	6.7	7.2
11	3	7.000	0.2646	0.1528	6.8	7.3
12	3	6.967	0.3055	0.1764	6.7	7.3
13	3	7.133	0.2517	0.1453	6.9	7.4
14	3	6.633	0.3055	0.1764	6.3	6.9
15	3	6.700	0.3606	0.2082	6.4	7.1
Total	45	6.978	0.3509	0.0523	6.3	7.8

Note: The standard prepared convergence angle was 6°.^22^,^23^,^24^

**Table 4:** One-way analysis of variance of the convergence angle measurements.

	Sum of Squares	dof	Mean Square	F	P value
Between Groups	3.044	14	0.217		
Within Groups	2.373	30	0.079	2.749	0.010
Total	5.418	44			

dof, degrees of freedom.
